# 3D-QSAR study of steroidal and azaheterocyclic human aromatase inhibitors using quantitative profile of protein–ligand interactions

**DOI:** 10.1186/s13321-017-0253-8

**Published:** 2018-01-18

**Authors:** Sehan Lee, Mace G. Barron

**Affiliations:** Gulf Ecology Division, U.S. Environmental Protection Agency, 1 Sabine Island Drive, Gulf Breeze, FL 32561 USA

**Keywords:** Aromatase inhibitor, Adverse outcome pathway, 3D-QSAR, Steroid, Azaheterocycle, Hydrophobic contact, Nitrogen–heme–iron coordination, Dual descriptor

## Abstract

Aromatase is a member of the cytochrome P450 superfamily responsible for a key step in the biosynthesis of estrogens. As estrogens are involved in the control of important reproduction-related processes, including sexual differentiation and maturation, aromatase is a potential target for endocrine disrupting chemicals as well as breast cancer therapy. In this work, 3D-QSAR combined with quantitative profile of protein–ligand interactions was employed in the identification and characterization of critical steric and electronic features of aromatase-inhibitor complexes and the estimation of their quantitative contribution to inhibition potency. Bioactivity data on pIC_50_ values of 175 steroidal and 124 azaheterocyclic human aromatase inhibitors (AIs) were used for the 3D-QSAR analysis. For the quantitative description of the effects of the hydrophobic contact and nitrogen–heme–iron coordination on aromatase inhibition, the hydrophobicity density field model and the smallest dual descriptor Δ*f*(r)^*S*^ were introduced, respectively. The model revealed that hydrophobic contact and nitrogen–heme–iron coordination primarily determines inhibition potency of steroidal and azaheterocyclic AIs, respectively. Moreover, hydrogen bonds with key amino acid residues, in particular Asp309 and Met375, and interaction with the heme–iron are required for potent inhibition. Phe221 and Thr310 appear to be quite flexible and adopt different conformations according to a substituent at 4- or 6-position of steroids. Flexible docking results indicate that proper representation of the residues’ flexibility is critical for reasonable description of binding of the structurally diverse inhibitors. Our results provide a quantitative and mechanistic understanding of inhibitory activity of steroidal and azaheterocyclic AIs of relevance to adverse outcome pathway development and rational drug design.

**Electronic supplementary material:**

The online version of this article (10.1186/s13321-017-0253-8) contains supplementary material, which is available to authorized users.

## Background

Aromatase cytochrome P450 is a key enzyme that catalyzes the rate-limiting step of aromatization in the biosynthesis of C18 estrogens from C19 androgens [1]. Deficiencies or excesses of estrogens are associated with various pathological states, thus over the last 10 years numerous toxicological and pharmacological studies have been devoted to identify and design aromatase inhibitors (AIs) [2–4]. Many endocrine-disrupting chemicals (EDCs) interfere with the endocrine system in humans and wildlife by modulation of aromatase activity, which can dramatically alter the rate production and disturb cellular and systemic levels of estrogen, ultimately leading to cancers, diabetes, or developmental problems [5]. In response to these significant adverse effects of EDCs on public and environmental health, the US Environmental Protection Agency (US EPA) Office of Research and Development (ORD) identified EDCs as one of its top six research priorities in 1996. In the same year, screening and testing for endocrine active chemicals was mandated under 1996 amendments to the Safe Drinking Water Act and Food Quality Protection Act [6]. To implement the legislation, the US EPA is developing adverse outcome pathways (AOPs) linking aromatase inhibition with adverse outcomes relevant to regulatory decision-making [7, 8].

Pathologically, estrogen promotes the growth and survival of breast cancer cells by binding and activating the estrogen receptor. The most direct breast cancer therapy is to reduce the amount of estrogen by interfering with its production via use of AIs. Because of their effectiveness, these AIs are quickly becoming the most frequently used anti-hormonal treatment for breast cancer. Further, some AIs are now being tested in breast cancer prevention trials [9, 10].

Chemicals typically initiate their therapeutic and adverse effects by binding to specific proteins through protein–ligand interactions. Therefore, a detailed understanding of the protein–ligand interactions is a central topic in the understanding biology at the molecular level as well as screening and design of active compounds. X-ray crystal structures of human aromatase in complex with the natural aromatase substrate androstenedione (4-androstene-3,17-dione, AD) and 6-substituted 1,4-androstadiene-3,17-diones (ADDs) have provided insights into the structural factors contributing to the catalytic and inhibitory mechanisms [1, 3, 11]. The ligands bind with their β-face oriented towards the heme group and C19 carbon within 4.3 Å from the iron atom. The Asp309 side chain and Met374 backbone amide that form hydrogen bond interactions with 3- and 17-keto oxygens, respectively, and the hydrophobic residues that pack tightly against the steroid backbone provide the molecular basis for the exclusive androgenic specificity of aromatase. C4 and C6 are near the active site access channel that begins at the protein-lipid bilayer interface, and long chain substituents at the 6β-position protrude into the access channel cavity.

AIs act through two distinct mechanisms to inhibit the action of aromatase and thereby reduce estrogen production [9]. Type I inhibitors such as atamestane, exemestane, and formestane are analogues of AD that bind competitively but irreversibly to the substrate-binding site of aromatase, causing permanent inactivation of the enzyme. Type II inhibitors such as letrozole, fadrozole, and vorozole are nonsteroidal compounds that interact reversibly with the heme prosthetic group of aromatase and occupy its substrate-binding site.

In the past decade, quantitative structure–activity relationship (QSAR) approaches based on 2D and 3D descriptors, pharmacophore, and molecular docking have been developed to predict inhibition potency of a limited number of structurally similar aromatase inhibitors [12–14]. However, critical protein–ligand interactions and their quantitative contribution to inhibition potency are still largely uncharacterized for broader groups of AIs, in particular for the hydrophobic contact and coordination to the heme–iron in the active site. In this study, a 3D-QSAR analysis of large number of steroidal and azaheterocyclic AIs elucidates the mechanisms of aromatase inhibition through identification and characterization of critical protein–ligand interactions in aromatase-inhibitor complexes and provides quantitative estimates of the contribution of each interaction to inhibition potency. A mechanistic understanding of aromatase-ligand interactions will facilitate development of AOPs and rational drug design for a diversity of AIs.

## Methods

### Dataset development

A dataset of chemical structures and in vitro inhibitory activities of human aromatase inhibitors was compiled following an exhaustive literature search and review. The in vitro activities were measured under similar experimental conditions using human placental microsomes incubated with 1*β*[^3^H]-androstenedione. Racemic mixtures and compounds containing highly flexible chain substituents (chain length ≥ 7) were excluded during dataset development resulting in 175 steroidal and 124 aromatic azaheterocyclic AIs. The in vitro activities were expressed as the half maximal inhibitory concentration (IC_50_) and transformed into corresponding pIC_50_ [− log(IC_50_)] as the expression of inhibition potency. The activity among the steroidal and azaheterocyclic AIs covered over three (42–200,000 nM) and four (1–467,000 nM) orders of magnitude for aromatase inhibition, respectively. The AIs in the dataset were protonated and energy minimized with MMFF94x using MOE (Molecular Operating Environment, Chemical Computing Group, Ontario, Canada). The structures, inhibition potencies, and references of the compounds are available in Additional file 1.

### Model development

**Fig. 1:**
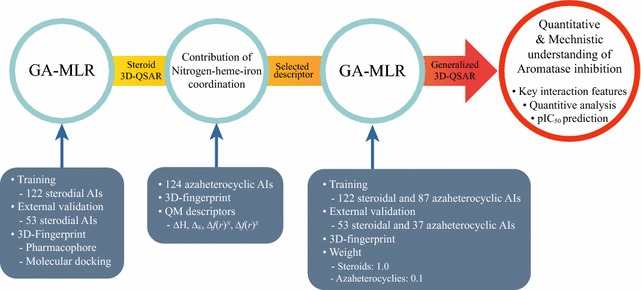
Description of 3D-QSAR development process for steroid and azaheterocyclic aromatase inhibitors

Both steroid-specific and generalized 3D-QSAR models were developed to account for different mechanisms of aromatase inhibition induced by steroidal and azaheterocyclic AIs. The steroid 3D-QSAR model development used the steroidal AIs and followed an iterative process with three stages: fingerprint generation, QSAR development, and pharmacophore refinement [15–17]. The fingerprint generation stage built 3D-fingerprints using molecular docking and a structure-based pharmacophore, then the 3D-QSAR model was trained with the generated fingerprint descriptors. At the third stage the pharmacophore was refined by adjusting its geometric parameters including distances and angles. The procedure was then repeated until no improvement in the mean absolute error (MAE) could be observed. The steroid 3D-QSAR model was then used to estimate the quantitative contribution of nitrogen–heme–iron coordination on aromatase inhibition by subtracting contributions of other interactions from the experimental pIC_50_ to develop a descriptor describing the heme coordination. The generalized 3D-QSAR model was built based on the steroidal and azaheterocyclic AIs with the developed heme coordination descriptor. The overall procedure is depicted in Fig. 1 and detailed below.

### Molecular docking

Docking experiments were conducted with ICM-Pro 3.8 [18]. For the proper representation of protein flexibility upon ligand binding, the flexible docking was performed with two human placental aromatase structures (PDB ID: 3S79 and 4GL7) [3], in which a set of residues remain flexible during docking process. The aromatase structures were downloaded from Protein Data Bank (RCSB PDB, http://www.rcsb.org) and prepared by removing water and ligand molecules from the PDB files. Formal charges of + 3.0, − 0.5, and − 1.0 were assigned to the heme–iron, four heme nitrogens, and Cys437 sulfur, respectively. The carboxylate of Asp309 was protonated before docking simulations. The ligand binding pocket for docking was defined by the active site residues (Arg115, Ile133, Phe134, Phe221, Trp224, Leu228, Ile305, Ala306, Asp309, Thr310, Val370, Leu372, Val373, Met374, Ile395, Ile398, Leu477, and Ser478) and heme prosthetic group.

### Bioactive conformation selection

For more thorough search of conformational space, ten independent docking simulations were performed on each protein–ligand complex. Among a large number of docked conformations generated by the repeated docking simulations, the conformations observed three or more times (RMSD < 0.5 Å) were used as candidates of the bioactive conformation to maximize the reproducibility of the results and reduce false positives of low probability. A bioactive conformation of a ligand among the candidate conformations was selected using a scoring function Δ*G*

1 $$\Delta G = {\text{pIC}}_{50}^{cal} + log\,S\left( r \right)$$

where ${\text{pIC}}_{50}^{cal}$ is the pIC_50_ estimated with a 3D-QSAR model. The steric hindrance *S*(*r*) of ligand with the active site residues was calculated using Lennard-Jones potential *U*(*r*) from AMBER force field [19]

2 $$S\left( r \right) = \sum\limits_{i}^{{N_{L} }} {\sum\limits_{j}^{{N_{R} }} {U\left( {r_{ij} } \right) } }$$

where *N*_*L*_ and *N*_*R*_ are the number of atoms in a ligand and the active site residues, respectively. In this work, only remarkable steric hindrances (*U*(*r*) ≥ 10) were taken into account.

### Structure-based pharmacophore model and 3D-fingerprint

Protein–ligand interaction features were identified using a structure-based pharmacophore approach, beginning with a search for common steric and electronic features observed in docked conformations. A fingerprint was generated to describe 3D protein–ligand interactions in the active site of aromatase. The docked conformations of inhibitors were mapped onto the developed pharmacophore and transformed into a 3D-fingerprint. Each bit of the 3D-fingerprint represents a pharmacophore feature.

### Hydrogen bond and interaction with the heme–iron

The pharmacophore features describing hydrogen bonds, interactions of 19-hydroxyl and 19-keto oxygens with the heme–iron, and nitrogen–heme–iron coordination were identified using a function of hydrogen bond term in GOLD [20], which is the product of three block functions.

3 $$\Delta R = B\left( \Delta r ,\Delta r_{ideal} ,\Delta r_{\text{max}} \right)B\left( \Delta \alpha ,\Delta \alpha_{ideal} ,\Delta \alpha_{\text{max}} \right)B\left( \Delta \beta ,\Delta \beta_{ideal} ,\Delta \beta_{{\text{max}}} \right)$$

A block function is defined as follows:

4 $${\text{B}}\left( x,x_{\text{ideal}} ,x_{ \text{max} } \right) = \left\{ {\begin{array}{*{20}l} 1 \hfill & {\text{if}}\,{{{x}} \le x_{\text{ideal}} } \hfill \\ {1.0 - \frac{{{{x - x}}_{\text{ideal}} }}{x_{ \text{max} } - x_{\text{ideal}} }} \hfill & {\text{if}}\,{x}_{\text{ideal}} \le x \le x_{ \text{max} } \hfill \\ 0 \hfill & {\text{if}}\,{{x}} > x_{ \text{max}} \hfill \\ \end{array} } \right.$$

where *r*, *α*, and *β* are ideal values for hydrogen-acceptor distance (H···A), donor-hydrogen-acceptor angle (D–H···A), hydrogen-acceptor-heavy atom attached to the acceptor angle (DH···A–X), respectively. *x*, *x*_*ideal*_, and *x*_*max*_ in the block function are the absolute deviation of an actual variable from the ideal value, the tolerance window around the variable within which the hydrogen bond is regarded as ideal, and the maximum possible deviation from the ideal value, respectively. For the interactions with the heme–iron, the heme–iron and Cys437 sulfur were labeled as H and D, respectively, and 19-hydroxyl and 19-keto oxygens and an aromatic azaheterocyclic nitrogen were labeled as A. A fingerprint bit for an interaction is 1, which means an aromatase-inhibitor complex forms the interaction, if Δ*R* is greater than or equal to 0.6. The interaction between a C19 carbon and the heme–iron is defined by distance between the atoms, whose bit is 1 if the distance is less than 4.3 Å.

### Hydrophobic contact interactions

An empirical hydrophobicity density field model was applied to measure the hydrophobic interactions between ligand and hydrophobic residues in the active site of aromatase. The hydrophobicity density at grid points on solvent accessible surface of ligand was calculated using generalized-solvation free energy density (G-SFED) model [21], and the hydrophobic contact (log *P*_*C*_) was obtained by integrating the hydrophobicity densities on the contact surface. Additional details of the method can be found in our previous study of estrogen receptor α [17].

## 3D-QSAR development

Multiple linear regression combined with genetic algorithm (GA-MLR) was carried out using the RapidMiner5.2 tool (http://rapid-i.com) to select important interaction features and analyze their quantitative contributions to aromatase inhibition. The model was built on a randomly selected set of 122 steroidal and 87 azaheterocyclic AIs (70% of the dataset) and validated using leave-one-out method and an external test set of the remaining 53 steroidal and 37 azaheterocyclic AIs. Due to the uncertainty of the binding mode of azaheterocyclic AIs and the limited understanding of the nitrogen–heme–iron coordination, weight values (steroid = 1.0 and azaheterocycle = 0.1) were used during the machine learning process.

### Nitrogen–heme–iron coordination

Four quantum mechanical descriptors, including enthalpy of formation of complex heme-azaheterocycle Δ*H* [22], the energy gap between highest occupied molecular orbital (HOMO) and lowest unoccupied molecular orbital (LUMO) Δ_*E*_, dual descriptor [23] of an aromatic azaheterocyclic nitrogen Δ*f*(*r*)^*N*^ which coordinate the heme–iron, and the smallest dual descriptor within the aromatic azaheterocycle Δ*f*(*r*)^*S*^ were calculated to describe the effects of nitrogen–heme–iron coordination on inhibition potency of azaheterocyclic AIs. All the calculations were done using Gaussian 03 W [24] and Multiwfn software [25]. The B3LYP functional was used with the LANL2DZ basis set with effective core potential on iron and the 3–21G basis set on all other elements to calculate Δ*H*. Δ_*E*_, Δ*f*(*r*)^*N*^, and Δ*f*(*r*)^*S*^ were calculated by B3LYP functional with 6–311 ++G(d,p) basis set. The optimized compound structures were obtained at HF/3-21G level of theory.

## Results

### Incorporation of protein flexibility in docking experiments

Proper representation of protein flexibility played a central role in determining binding poses and affinities of the steroidal AIs with a structurally diverse pattern of substituents at 2-, 3-, 4-, 6-, 7-, 10-, 16-, 17-, and 19-positions. The protein flexibility was incorporated in the molecular docking by the use of an ensemble consisting of two human placental aromatase structures. A residue, Phe221 or Thr310, which allowed the rigid steroid core to bind in the conserved manner observed in the crystal structures, was treated as flexible during the docking for the steroidal AIs. Phe221 is located at the entrance of access channel and undergoes a rotation to provide sufficient space for the steroids with a bulky (more than two heavy atoms) 2-, 2α-, 4-, 6-, or 6α-substituent and estrogen derivatives. 4α-substituted steroids were not found in the data set, but it is likely that a bulky 4α-substituent could be accommodated in the access channel by the conformational changes of Phe221. Thr310 also provides space for bulky 4β- and 6β-substituents by changing its side chain dihedral angle. Due to the absence of aromatase crystal structures in complex with azaheterocycles and structural diversity in azaheterocyclic AIs, the docking experiments for azaheterocyclic AIs were performed using the rigid aromatase structures.

### Structure-based pharmacophore and 3D-fingerprint

The structure-based pharmacophore captured both geometric and electronic features common to the bioactive conformations and included 11 candidate features: (1) a hydrogen bond donor that interacts with the carbonyl oxygen of Ala306, (2) a hydrogen bond acceptor that interacts with the protonated Asp309 side chain, (3) a hydrogen bond acceptor that interacts with the Thr310 side chain, (4) a keto or ether oxygen that form a hydrogen bond with the amide proton of Met374, (5) a hydroxyl oxygen that form a hydrogen bond with the amide proton of Met374, (6) a nitro oxygen that form a hydrogen bond with the amide proton of Met374, (7) a nitrile nitrogen that form a hydrogen bond with the amide proton of Met374, (8) an aromatic nitrogen that form a hydrogen bond with the amide proton of Met374, (9) a 19-hydroxy or 19-oxo oxygen or a C19 carbon that interacts with the heme–iron, (10) an aromatic azaheterocyclic nitrogen that coordinates the heme–iron, and (11) hydrophobic contact (log *P*_*C*_) with hydrophobic residues in the active site. The determined block function parameter values and their meanings (Eqs. 3, 4) are summarized in Table 1. The features 7, 8, and 10 were observed only in the aromatase-azaheterocycle complexes.

**Table 1:** Values and meanings of block function parameters for identification of protein–ligand interaction features

Term	Meaning	Value		
		Hydrogen bond	Interaction with heme–iron	
			C19–OH or = O	Azaheterocycle
H···A distance parameters (Å)				
*r*	The ideal H···A distance	2.00	2.40	2.25
Δ*r*_*ideal*_	The tolerance window around *r*, with in which the hydrogen bond is regarded as ideal	0.50	0.25	0.25
Δ*r*_*max*_	The maximum possible deviation from *r*; above this, the interaction is not regarded as a hydrogen bond	0.65	0.65	0.65
D–H···A angle parameters (°)				
*α*	The ideal D–H···A angle	180	180	180
Δ*α*_*ideal*_	The tolerance window around *α*, with in which the hydrogen bond is regarded as ideal	45	10	20
Δ*α*_*max*_	The maximum possible deviation from *α*; above this, the interaction is not regarded as a hydrogen bond	80	40	40
DH···A–X angle parameters (°)				
*β*	The ideal DH···A–X angle	180	180	180
Δ*β*_*ideal*_	The tolerance window around *β*, with in which the hydrogen bond is regarded as ideal	80	80	60
Δ*β*_*max*_	The maximum possible deviation from *β*; above this, the interaction is not regarded as a hydrogen bond	100	100	100

*H* hydrogen, *D* donor, *A* acceptor, *X* heavy atom attached to A

## 3D-QSAR for understanding inhibition potency

Two 3D-QSAR models were developed: (1) a steroid 3D-QSAR model for developing a descriptor describing the nitrogen–heme–iron coordination, and (2) a generalized 3D-QSAR model for identifying key steric and electronic features and analyzing their quantitative contribution to inhibition potency of structurally diverse steroidal and azaheterocyclic AIs with different inhibition mechanisms. The optimal generalized 3D-QSAR model had the nine bits fingerprint: seven binary bits for six hydrogen bonds and an interaction with the heme–iron (FP1-FP7) and two continuous bits for nitrogen–heme–iron coordination and log *P*_*C*_ (FP8 and FP9). Hydrogen bonds of hydroxyl oxygen and nitro oxygen with the amide proton of Met374 were not selected due to their low contributions. A summary of the developed pharmacophore, fingerprint, and 3D-QSAR models is provided in Table 2.

**Table 2:** Summary of pharmacophore, fingerprint, and QSAR models parameters

Pharmacophore			Fingerprint Index	QSAR coefficient	
Type	Amino acid	Ligand^a^		Steroid	Generalized
Hydrogen bond	Ala306	Any donor	FP1	0.296	0.229
Hydrogen bond	Asp309	Any acceptor	FP2	0.679	0.621
Hydrogen bond	Thr310	Any acceptor	FP3	0.791	0.710
Hydrogen bond	Met374	Keto or ether oxygen	FP4	0.823	0.821
Hydrogen bond	Met374	Nitrile nitrogen	FP5	NA	1.278
Hydrogen bond	Met374	Nar^c^ nitrogen	FP6	NA	2.237
Heme–iron interaction	Heme–iron	19-OH, 19 = O, C19	FP7	0.721	0.724
Coordination	Heme–iron	Nar^c^	FP8	NA	38.587Δ*f*(r)^*S*^ + 3.931
log *P*_*C*_	Hydrophobic residues	Hydrophobic surface	FP9	2.234	1.969
Intercept	NA^b^	NA	NA	0.270	0.755

^a^Nitro group was excluded

^b^Not applicable

^c^Aromatic azaheterocycle

**Fig. 2:**
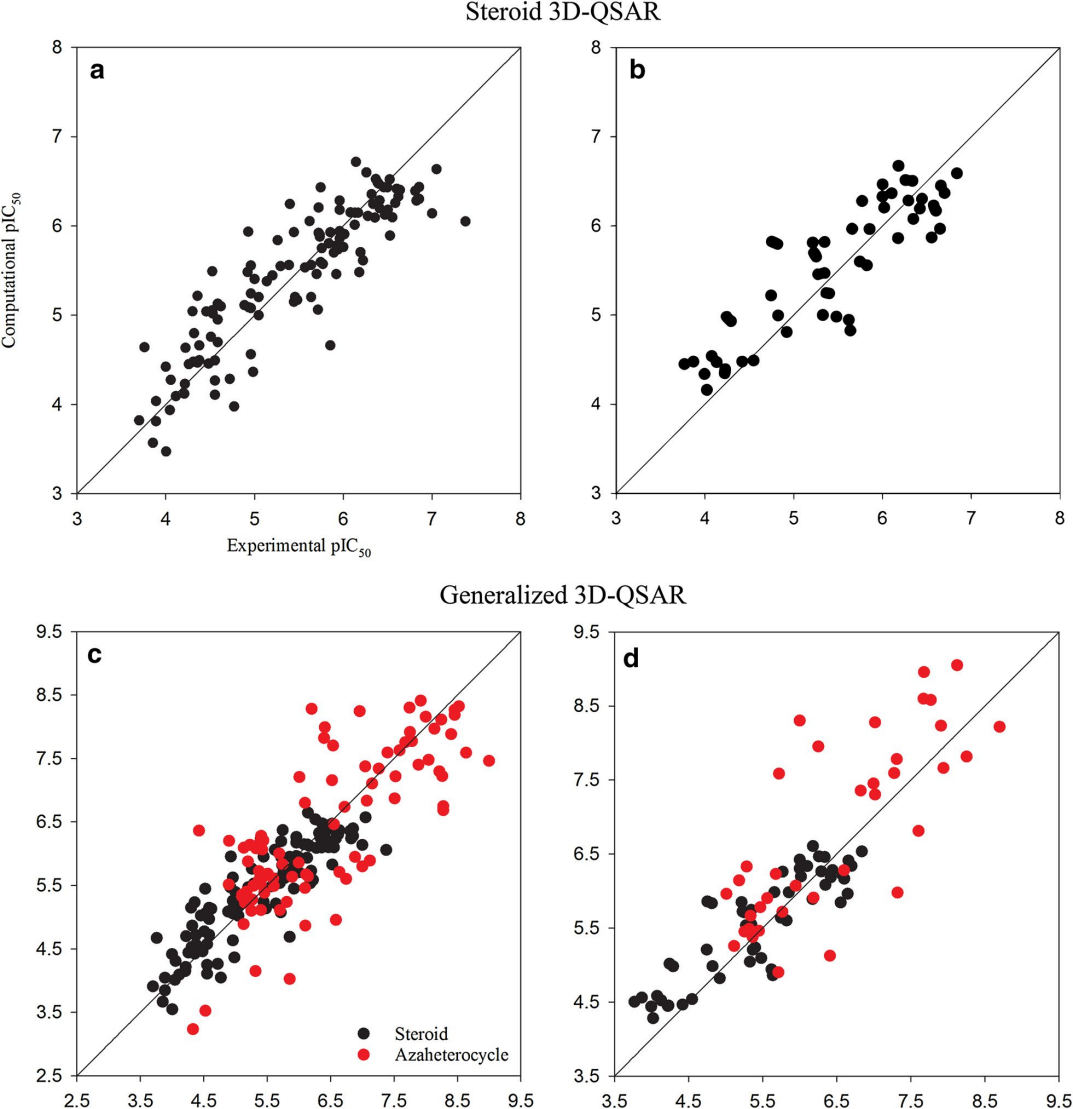
Scatter plots of pIC_50_ calculated with steroid (**a**, **b**) and generalized (**c**, **d**) 3D-QSAR models for the training sets (**a**, **c**) and external validation sets (**b**, **d**)

As shown in Table 3, the steroid 3D-QSAR model exhibited significant self-consistency (R^2^ = 0.78) as well as high internal predictive ability (Q^2^ = 0.76). External validation of the model with a set of 53 steroids resulted in R^2^ of 0.77. Most of the steroids (136, 78 percent) were predicted within a 0.5 log unit error, and only four steroids had prediction errors between 1.0 and 1.4 log units. The generalized 3D-QSAR model showed lower but acceptable performance, where R^2^ and MAE for training set were 0.73 and 0.449 log units, respectively. The results of leave-one-out cross (Q^2^ = 0.75) and external validations (R^2^ = 0.72) demonstrated good predictive power of the generalized model. Plots of the computational results versus the experimental pIC_50_ are shown in Fig. 2. The 3D-fingerprints and predicted pIC50 values are available in Additional file 1.

**Table 3:** Performance of the steroid and universal 3D-QSAR models

Data set	Value	Steroid	Generalized		
			Whole	Steroid	Azaheterocycle
Training	Data no.	122	209	122	87
	R^2^	0.78	0.73	0.78	0.60
	Q^2^	0.76	0.75^a^	NA^b^	NA
	MAE	0.326	0.449	0.328	0.619
External validation	Data no.	53	90	53	37
	R^2^	0.77	0.72	0.76	0.59
	MAE	0.365	0.496	0.377	0.667

^a^1.0 and 0.1 weight values were used for steroid and azaheterocycle data, respectively, during training process

^b^Not applicable

### Description of nitrogen–heme–iron coordination

**Fig. 3:**
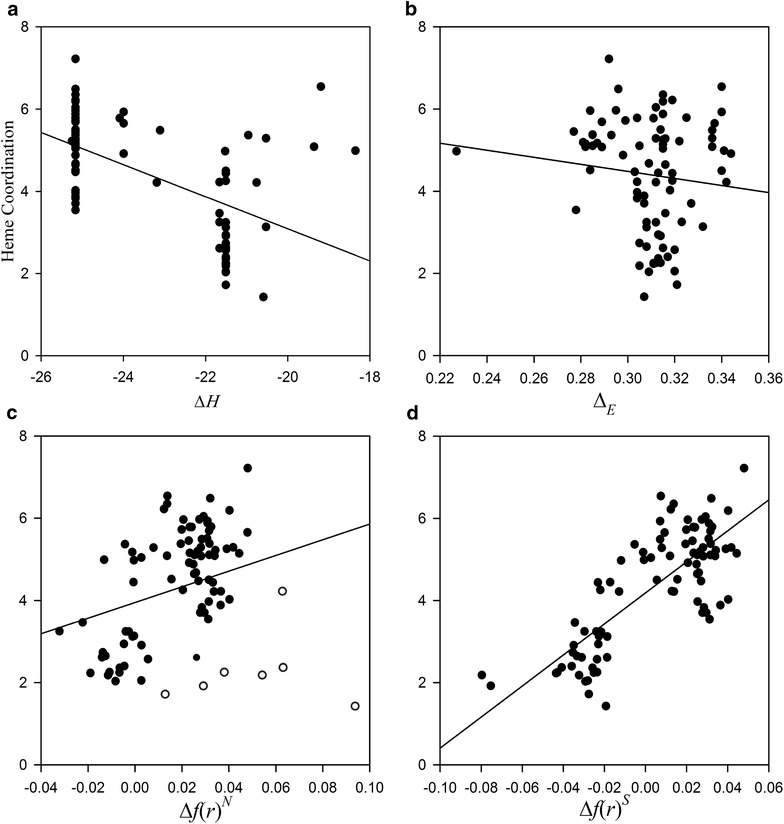
Correlation of quantum mechanical descriptors, enthalpy of formation (Δ*H*, **a**), HOMO-LUMO gap (Δ_*E*_, **b**), dual descriptor (Δ*f*(*r*)^*N*^, **c**), and smallest dual descriptor (Δ*f*(*r*)^*S*^, **d**), with the contribution of nitrogen–heme–iron coordination to inhibition potency. The eight outliers are shown as open cycles (**c**)

The azaheterocycles that coordinate with the heme–iron were identified using the scoring function (Eq. 1) and subjected to analysis of the nitrogen–heme–iron coordination. Docked conformations forming the coordination were generated for 104 out of 124 azaheterocyclic AIs, and 87 of the conformations were selected as the bioactive conformation. Density functional theory (DFT) calculations were performed on the different azaheterocyclic groups, including 1,2,3-triazole, 1,2,4-triazole, imidazole, isoquinoline, phthalazine, pyrazole, pyridazine, pyridine, pyrimidine, and tetrazole, to determine Δ*H* of each group of compounds. The results showed that Δ*H* (Fig. 3a) and Δ_*E*_ (Fig. 3b) could not sufficiently describe the coordination of azaheterocyclic AIs, where R^2^ values were 0.30 and 0.0, respectively.

The dual descriptor is a local reactivity descriptor defined as the difference between the nucleophilic and electrophilic Fukui functions

5 $$\Delta f\left( r \right) = f^{ +} \left( r \right) - f^{ - } \left( r \right)$$

If Δ*f*(*r*) > 0, then the site is favored for a nucleophilic attack, whereas if Δ*f*(*r*) < 0, then the site may be favored for an electrophilic attack. Δ*f*(*r*)^*N*^ showed low correlation (R^2^ = 0.08) but could describe the coordination well (R^2^ = 0.41) excluding eight outliers which far overestimate the heme coordination (Fig. 3c). The dual descriptor was modified in different ways to develop more informative descriptor that can explain the coordination well. The smallest dual descriptor of an atom within the aromatic azaheterocycle Δ*f*(*r*)^*S*^ showed high correlation with the coordination (R^2^ = 0.61) (Fig. 3d) and was used for development of the generalized 3D-QSAR model.

## Discussion

### Protein flexibility in ligand binding

**Fig. 4:**
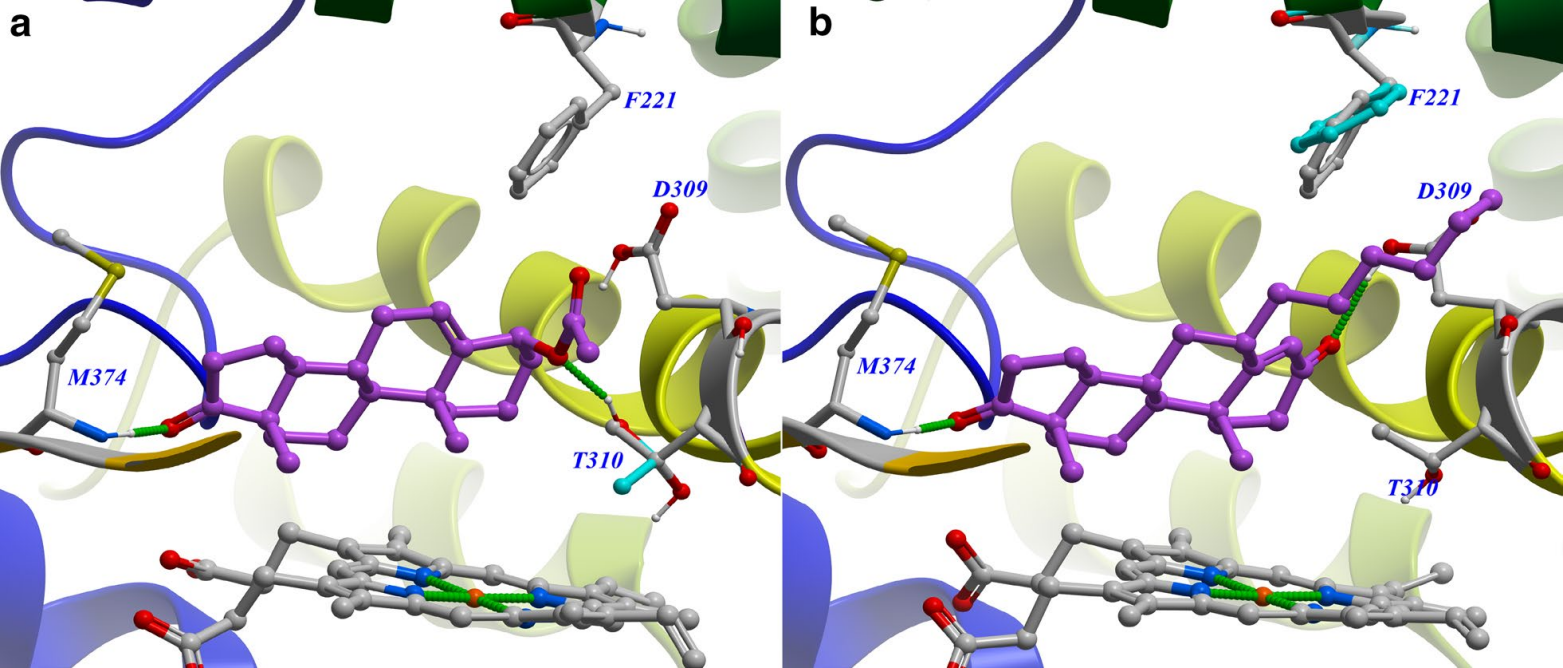
Close-up view of the aromatase active site in complex with 4β-acetoxy 5-androstene-17-one (**a**) and 6α-n-hexyl 4-androstene-3,17-dione (**b**). The protein backbone is rendered in rainbow color (N terminus, blue; C terminus, red): carbon, gray; nitrogen, blue; oxygen, red; iron, orange. The ligand carbons are shown in magenta and optimized flexible Thr310 (**a**) and Phe221 (**b**) residues are shown in cyan. The hydrogen bonds between the ligands and active site residues are drawn as green dashed lines

A complete and conclusive understanding of aromatase inhibition has remained elusive because of limited understanding of conformational changes of aromatase upon ligand binding and the effects of interactions with the active site and the heme–iron on ligand affinities [26–28]. Ligand binding can involve a wide range of induced conformational changes in the protein backbone and side chains to form specific protein–ligand complex. It is therefore critical to accurately take into account the protein flexibility in ligand docking and virtual screening [29, 30]. The crystal structures of human placental aromatase showed that most residues in the active site were inflexible, adopting similar conformations in the crystal structures, but the side chain dihedral angle of Thr310 varied up to 53° to reduce steric hindrance and maintain a hydrophobic contact with the 6β-2-alkynyloxy groups accommodated in the access channel. Upon inspection of the flexible docking results, it was observed that binding modes of 4β-, 6β-, 4-, 6-, 6α-substituted androgens are similar with crystal binding modes of the 6β-2-alkynyloxy ADDs. The 4β- and 6β-substituents were accommodated in the access channel and the side chain dihedral angle of Thr310 varied up to 167° to reduce steric hindrance and stabilize the complexes. Specifically, Thr310 stabilized the 4β-acetoxy 5-androstene-17-one by forming hydrogen bond with the acetoxy group (Fig. 4a). On the other hand, 4-, 6-, and 6α-substituents protruded into the access channel which induces conformational changes in the Phe221 side chain to reduce steric hindrance and maintain a hydrophobic contact with the substituents (Fig. 4b).

### Hydrophobic contacts

Inhibition potency was expressed as a linear combination of interaction features

6 $${\text{pIC}}_{50} = \sum\nolimits_{i = 1}^{10} {c_{i} {\text{FP}}_{i} + C} .$$

**Fig. 5:**
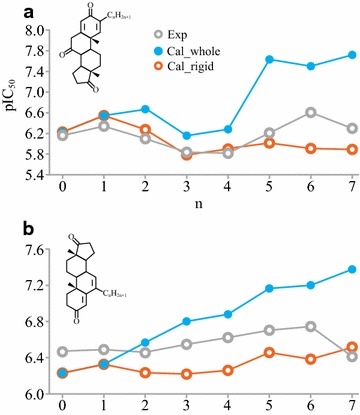
Comparison of experimental pIC_50_ values of 2-n-alkyl 1,4-androstadiene-3,7,17-trione (**a**) and 6-n-alkyl 4,6-androstadiene-3,17-dione (**b**) with computational values. The pIC_50_ values were calculated with (blue) or without (orange) the hydrophobic contact of the n-alkyl chain. n is the number of carbon in alkyl chains

The product of a bit in the 3D-fingerprint, FP_*i*_, and its regression coefficient, *c*_*i*_, represents the independent contributions of each interaction feature to inhibition potency. The intercept *C* is the inhibition potency without any protein–ligand interactions, which is approximately zero in the both 3D-QSAR models. The importance of a hydrophobic character for the aromatase inhibition has been well recognized [31–33], but there are no theoretical or experimental studies for estimating the quantitative contribution from the hydrophobic contact. In this study, the log *P*_*C*_ describing the hydrophobic interactions was calculated by the sum of hydrophobicity densities on the hydrophobic contact surface. The hydrophobic core of steroids extensively interacted with hydrophobic residues including Ile133, Phe134, Phe221, Trp224, Val370, and Leu477 and this observation is in agreement with previous reports [1, 34]. Diverse flexible substituents at different positions also formed hydrophobic contact, but the inclusion of these hydrophobic contacts resulted in overestimation of inhibition potency (Fig. 5a). This observation is consistent with our previous results that without steric hindrance or a hydrogen bond to reduce degree of rotational freedom a flexible group can adapt alternate conformations which destabilizes the hydrophobic contacts and reduces binding affinity [15, 17]. 4-, 4β-, 6-, 6α-and 6β-substituents accommodated in the accessible channel formed extensive hydrophobic interactions with Thr310, Phe221, Val369, Val370, Ser478, but could not contribute to inhibition potency (Fig. 5b). Therefore, atoms in the flexible substituents and access channel were excluded in log *P*_*C*_ calculation for both steroidal and azaheterocyclic AIs.

### Inhibition potency of steroidal AIs

The results of 3D-QSAR models indicates that inhibition potency of steroidal AIs is markedly dependent on the hydrophobic nature of steroid core and potent steroidal AIs form hydrogen bonds with residues and interact with the heme–iron. In the generalized 3D-QSAR model, the calculated log *P*_*C*_ values for the 175 steroids ranged from 1.286 to 2.125 corresponding to from 2.533 to 4.185 orders of magnitude in pIC_50_, which account for up to 83 percent of the inhibition potency.

A hydroxyl, ether, or keto group could form a hydrogen bond with Ala306, Thr310, Asp309, and Met374 depending on position and configuration of the group and increase inhibition potency less than one order of magnitude (approximately from 2 to 7-fold). The 17-keto oxygen is responsible for a hydrogen bond contact with the amide backbone of Met374. Moreover, 3-keto, 3α-hydroxyl, 4-keto, and 4-hydroxyl oxygens in AD derivatives are found to form hydrogen bonds with the Asp309 side chain, whereas 3-hydroxyl in estrogen derivatives could form a hydrogen bond with the Ala306, Thr310, or Asp309. 4β-hydroxyl oxygen is found to form hydrogen bond with the Ala306. One steroidal and many azaheterocyclic AIs have a nitro group that forms a hydrogen bond with the Asp309 side chain or amide backbone of Met374, but contributions of the hydrogen bonds were negligible. This is consistent with the experimental evidence that the nitro group is a very poor hydrogen bond acceptor in contrast to the excellent hydrogen bonding capacity of the keto and carboxylic acid groups [35].

**Fig. 6:**
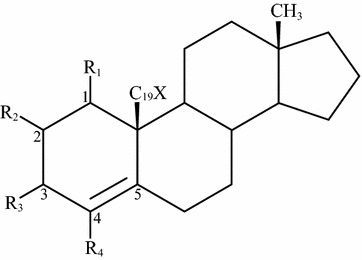
Scheme of steroid structure used to define interactions of 19 heteroatoms with the heme–iron. X is hydroxyl (OH) or oxo (= O). R1 and R2 are hydrogens. R3 is hydrogens or ketone. R4 is any functional group

The C19 carbon and 19-hydroxy and 19-oxo oxygens of androgens are positioned sufficiently close to the heme moiety to allow direct attack by an iron-bound oxidant [36]. Inspection of the steroid 3D-QSAR results for 15 available 19-hydroxy and 19-oxo derivatives indicates that only androgen derivatives with specific structures, which might be related to reactivity of the oxygens, are able to form sufficient interaction with the heme. Therefore, the interaction feature of 19-hydroxyl and 19-keto oxygens was identified by considering both binding geometry and environment of the C19 oxygens (Fig. 6). The interactions with the heme moiety contributed to 5.3-fold increase in inhibition potency.

### Inhibition potency of azaheterocyclic AIs

The results of the generalized 3D-QSAR suggest that high affinities of azaheterocyclic AIs arise from their dual interaction with the active site and the heme–iron. Most azaheterocyclic AIs were small compounds with highly polar groups, such as nitro and nitrile, together with at least one polar azaheterocycle. Therefore, the azaheterocyclic AIs form less hydrophobic contacts compared with steroidal AIs, where log *P*_*C*_ values for the 124 azaheterocyclic AIs ranged from 0.203 to 1.910 corresponding to from 0.400 to 3.762 orders of magnitude in pIC_50_, which account for approximately 10–50% of inhibition potency. Many azaheterocyclic AIs have nitrile groups and could form a hydrogen bond with the amide backbone of Met374 increasing inhibition potency 19-fold. Aromatic azaheterocyclic nitrogen also could form a hydrogen bond with the amide backbone of Met374 and significantly stabilized interaction with aromatase (173-fold increase in inhibition potency).

The coordination of aromatic azaheterocyclic nitrogen with the iron atom of the heme moiety is an important feature of potent and selective aromatase azaheterocyclic AIs [2, 37]. In an effort to determine an electronic feature important in binding besides the nitrogen–heme–iron coordination we attempted to develop a quantum–mechanical descriptor correlated with the contribution of the heme coordination. The contribution of the heme coordination was estimated indirectly by subtracting the contributions of the other interaction features from the experimental inhibition potency and ranged from 1.427 to 7.219 log units in pIC_50_. The significance and variance of the heme coordination urges the use of a numerical descriptor other than the binary, presence (1) or absence (0), for describing insignificant contributions (< 1 log unit) of hydrogen bonds and interactions with heme–iron (FP1-FP7). The quantum mechanical descriptors describing chemical reactivity Δ*H* and Δ_*E*_ have been successfully applied to describe aromatase inhibitory activity of structurally similar or simple azaheterocycles [38, 39] but could not explain the structurally diverse azaheterocycles of this study. The developed smallest dual descriptor Δ*f*(*r*)^*S*^ provided sufficient description of the coordination (R^2^ = 0.61) and indicates that the effects of nitrogen–heme–iron coordination on ligand affinity depends on minimal nucleophilic reactivity of an azaheterocycle rather than that of the azaheterocyclic nitrogen coordinating the heme–iron.

### Quantitative profile of aromatase-steroid interactions

**Fig. 7:**
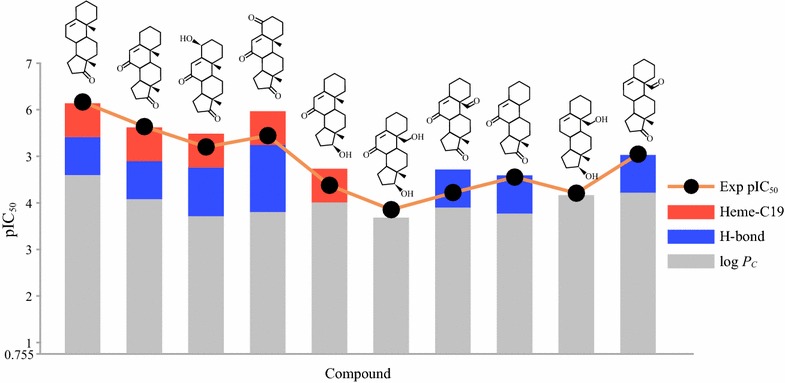
Prediction of pIC_50_ of 5-androstae-17-one derivatives. pIC50 is described by contributions from hydrophobic contacts (gray), hydrogen bonds (blue), and interaction with the heme–iron (red)

Introduction or elimination of a functional group in a ligand induces changes in steric and electronic properties that modify protein–ligand complex structure and bind affinity. The prediction results for the steroidal AIs showed that the generalized 3D-QSAR can successfully explain the pIC_50_ variation according to the structural modification. Introduction of a polar group, such as hydroxyl and ketone, at 3-, 4-, or 17-position resulted in formation a hydrogen bond with Ala306, Asp309, Thr310, or Met374, which accounts for from 0.229 to 0.821 orders of magnitude increase in pIC_50_, but also decrease in hydrophobicity of ligand around the substitution position. Introduction of polar groups at other positions decreased pIC50 by reducing hydrophobic contacts. The pIC_50_ variations in structural modification are shown in Fig. 7. Introduction of a keto group at 7-position of 5-androstene-17-one induced 1.016 orders of magnitude decrease in pIC_50_ by reducing log *P*_*C*_ near the 7-position. An additional 4*β*-hydroxyl or 4-keto group could form a hydrogen bond with Ala306 or Asp309 increasing pIC_50_ by 0.229 and 0.621 orders of magnitude, respectively, but also decrease log *P*_*C*_ by 0.364 and 0.274 corresponding to 0.718 and 0.539 order of magnitude in pIC_50_, respectively. Substitution of the 17-keto group in 5-androstene-7,17-dione with hydroxyl group resulted in loss of a hydrogen bond with Met374, which account for 0.821 orders of magnitude decrease in pIC_50_. The C19 demethylation and many of 19-hydroxyl and 19-keto substitutions resulted in loss of the interaction with the heme–iron and decrease in log *P*_*C*_ up to 0.325, which account for 0.724 and 0.640 orders of magnitude decrease in pIC_50_, respectively. These observations are consistent with the results of previous QSAR study [34] suggesting that the optimum number of hydrogen bond acceptor should be less than or equal to two and optimal hydrophobicity for ideal aromatase inhibitors.

## Conclusion

In this study, we have developed a framework for understanding inhibition mechanisms of steroid and azaheterocyclic AIs based on the 3D-QSAR approach combined with quantitative profile of protein–ligand interactions. The hydrophobicity density field model and the smallest dual descriptor Δ*f*(*r*)^*S*^ were successfully used in explaining stabilization of aromatase-inhibitor complex through the hydrophobic contact and nitrogen–heme–iron coordination, respectively. The results clearly show structural factors of potent steroidal and azaheterocyclic AIs: (1) hydrophobic steroid backbone with one or two hydrogen bond acceptors that form potent hydrogen bond with Asp309 or Met375 and C19 or C19 heteroatom that interact with the heme–iron and (2) highly reactive azaheterocycles with proper conformation that coordinate the heme–iron. Our approach represents a first step toward the in silico evaluation of aromatase inhibitory potency during the early stages of toxicity assessment, and will facilitate AOP development and breast cancer drug discovery.

## Additional file

**Additional file 1** Dataset for model development.

## Abbreviations

EDC: endocrine disrupting chemical
AI: aromatase inhibitor
EPA: Environmental Protection Agency
ORD: Office of Research and Development
AOP: adverse outcome pathway
AD: 4-androstene-3,17-dione
ADD: 1,4-androstadiene-3,17-dione
QSAR: quantitative structure–activity relationship
MAE: mean absolute error
RMSD: root-mean-square deviation
G-SFED: generalized-solvation free energy density
HOMO: highest occupied molecular orbital
LUMO: lowest unoccupied molecular orbital
